# Fall Armyworm, *Spodoptera frugiperda* Infestations in East Africa: Assessment of Damage and Parasitism

**DOI:** 10.3390/insects10070195

**Published:** 2019-07-03

**Authors:** Birhanu Sisay, Josephine Simiyu, Esayas Mendesil, Paddy Likhayo, Gashawbeza Ayalew, Samira Mohamed, Sevgan Subramanian, Tadele Tefera

**Affiliations:** 1 Plant Health Theme, International Center of Insect Physiology & Ecology (ICIPE), P.O. Box 5689, Addis Ababa, Ethiopia; 2 School of Pant Sciences, Haramaya University, P.O. Box 138, Dire Dawa, Ethiopia; 3 Melkassa Agricultural Research Centre, P.O. Box 436, Adama, Ethiopia; 4 Plant Health Theme, International Center of Insect Physiology & Ecology (ICIPE), P.O. Box 30772-00100 Nairobi, Kenya; 5 Department of Horticulture & Plant Sciences, Jimma University College of Agriculture & Veterinary Medicine, P.O. Box 307, Jimma, Ethiopia; 6 Kenya Agricultural & Livestock Research Organization, P.O. Box 5781, Nairobi, Kenya

**Keywords:** fall armyworm, *Telenomus remus*, *Cotesia icipe*, local parasitoid, maize

## Abstract

The fall armyworm (FAW), *Spodoptera frugiperda*, threatens maize production in Africa. A survey was conducted to determine the distribution of FAW and its natural enemies and damage severity in Ethiopia, Kenya and Tanzania in 2017 and 2018. A total of 287 smallholder maize farms (holding smaller than 2 hectares of land) were randomly selected and surveyed. FAW is widely distributed in the three countries and the percent of infested maize fields ranged from 33% to 100% in Ethiopia, 93% to 100% in Tanzania and 100% in Kenya in 2017, whereas they ranged from 80% to 100% and 82.2% to 100% in Ethiopia and Kenya, respectively, in 2018. The percent of FAW infestation of plants in the surveyed fields ranged from 5% to 100%. In 2017, the leaf damage score of the average of the fields ranged from 1.8 to 7 (9 = highest level of damage), while 2018, it ranged from 1.9 to 6.8. In 2017, five different species of parasitoids were recovered from FAW eggs and larvae. *Cotesia icipe* (Hymenoptera: Braconidae) was the main parasitoid recorded in Ethiopia, with a percent parasitism rate of 37.6%. *Chelonus curvimaculatus* Cameron (Hymenoptera: Braconidae) was the only egg-larval parasitoid recorded in Kenya and had a 4.8% parasitism rate. In 2018, six species of egg and larval parasitoids were recovered with *C. icipe* being the dominant larval parasitoid, with percentage parasitism ranging from 16% to 42% in the three surveyed countries. In Kenya, *Telenomus remus* (Hymenoptera: Scelionidae) was the dominant egg parasitoid, causing up to 69.3% egg parasitism as compared to only 4% by *C. curvimaculatus*. Although FAW has rapidly spread throughout these three countries, we were encouraged to see a reasonable level of biological control in place. Augmentative biological control can be implemented to suppress FAW in East Africa.

## 1. Introduction

Maize (*Zea mays* L.) is the most important staple food crop in Africa [1] and is predominantly grown by smallholder farmers. However, the production of this crop and consequently the livelihood of the growers is threatened by the invasion and widespread infestation of the fall army worm (FAW), *Spodoptera frugiperda* (J. E. Smith) (Lepidoptera: Noctuidae) which has led to substantial maize yield losses [2,3]. FAW was first reported in late 2016 in West Africa and it rapidly spread to different parts of the continent. Currently, its occurrence has been officially reported in 44 African countries [4,5]. In the Americas, there are two races of FAW, namely the rice strain (R-strain), which is most consistently found in millet and grass species associated with pasture habitats, whereas the corn strain (C-strain) prefers maize and sorghum. The two strains of FAW have also been reported in Africa [2,6,7,8]. FAW causes devastating damage to almost 100 plant species, including maize, sorghum, rice, soybean, cotton, wheat and sugarcane. On the other hand, the recent review by Montezano et al. [9] documented a total of 353 FAW larval host plant species belonging to 76 plant families, with the greatest number of host taxa in the family Poaceae (106 taxa), followed by Asteraceae and Fabaceae (31 taxa each). Due to its ability to rapidly spread and inflict widespread damage across multiple crops, FAW poses a serious threat to the food and nutrition security and livelihoods of millions of farming households in sub-Saharan Africa (SSA) [2,3,4,5].

In maize, FAW attacks all crop stages from seedling emergence through to ear development. They defoliate and can kill young plants, whorl damage can result in yield losses, and ear feeding can result in grain quality and yield reductions [10]. Detecting FAW infestations before it causes economic damage is the key to its management. If infestations are detected too late, the impacts of damage maybe irreversible [4,10]. Recent estimates by CABI in 12 maize-producing countries showed that without control, FAW can cause maize yield losses ranging from 4.1 to 17.7 million tonnes per year, which is equivalent to an estimated loss between US$ 1088 and US$ 4661 million annually [4]. Recently, Baudron et al. [11] reported yield loss of 11.57% due to FAW damage in smallholder maize fields in Zimbabwe, which is relatively lower than the perceived losses reported by smallholder farmers in different countries such as in Ghana and Zambia [2,4].

The common management strategy for the FAW in the Americas has been the use of insecticide sprays and genetically modified crops (Bt maize) [2]. Soon after the occurrence of FAW infestation, a massive spraying programme of chemical insecticides was deployed by governments of African countries [2,5]. However, most smallholder farmers in Africa cannot afford repeated sprays of insecticides and Bt maize is not available in Africa. Furthermore, excessive use of chemical insecticides removes potential natural enemies, negatively impacts human and livestock health, leads to resistance development in target pests and increases crop production costs [2,5,12]. In general, the excessive usage of insecticides and associated risks has raised food safety and sustainability concerns. This highlights the need for development of integrated pest management (IPM) strategies that suit the needs of the African smallholder farmers. Furthermore, FAW being arecent invader in the continent, information on natural enemies associated with this pest is not well-documented for Africa.

A wide range of natural enemies, including parasitoids, arthropod predators and entomopathogens attack FAW in its native region. For example, Molina-Ochoa et al. [13] listed about 150 species of parasitoids of FAW in the Americas and Caribbean. Some species of egg and larval parasitoids have been reported in East and West Africa [4]. For the development of IPM programs for FAW, it is imperative to determine its current distribution and the magnitude of damage it causes in maize-growing areas, and to develop an inventory of indigenous natural enemies that have made new associations with the pest. Therefore, the objectives of the present study were to assess the level of maize damage caused by FAW in Ethiopia, Kenya and Tanzania, and to determine the association of indigenous natural enemies with FAW.

## 2. Materials and Methods

### 2.1. Study Area Description

**Figure 1:**
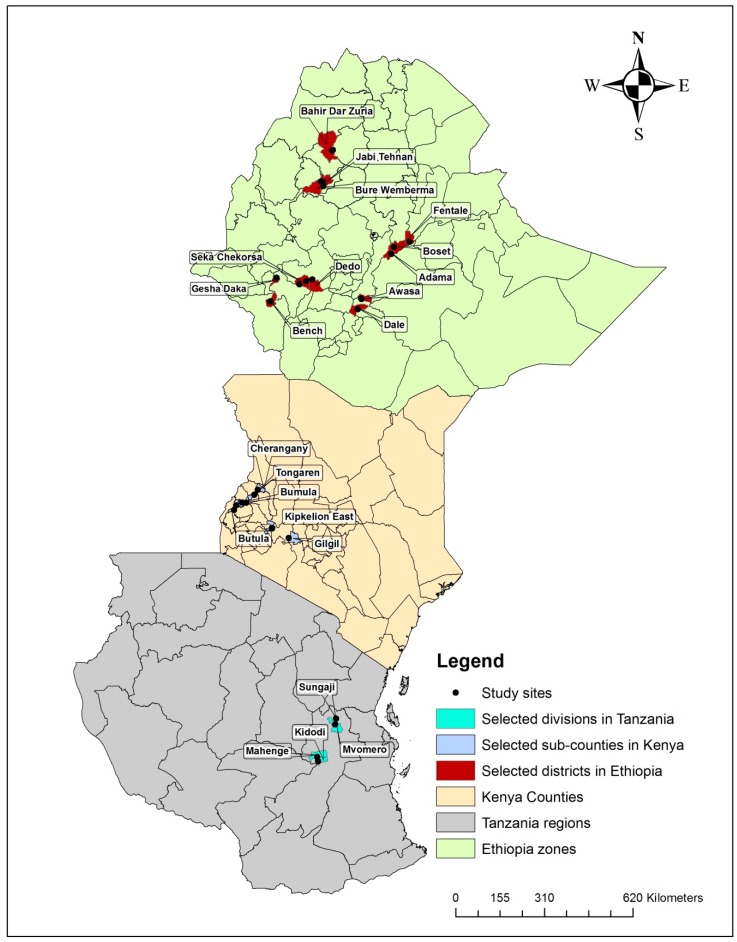
Map showing study districts in Ethiopia, Kenya and Tanzania.

Surveys of FAW were conducted in major maize-growing districts of Ethiopia, Kenya and Tanzania (Figure 1 and Table 1).

### 2.2. Damage Assessment

In the three countries surveyed, districts and farms were purposely selected based on reported occurrence of FAW. A total of 287 randomly selected maize farms were surveyed, with 188 farms in Ethiopia, 81 farms in Kenya and 18 farms in Tanzania. Surveys were conducted from March to October 2017 and June to August 2018 in Ethiopia, from April to August 2017 and June to August 2018 in Kenya and from July to November 2017 in Tanzania. In all districts, the surveys covered the growing period of maize one month after planting to harvest. In each surveyed farm, three quadrants measuring 3 m × 3 m were randomly selected and total number of plants and damaged plants were counted. Percent infested fields were calculated as follows:

%FAW infested fields = (Number of FAW infested fields)/(Total number of fields surveyed) × 100

Percent infested plants per quadrant was calculated using the formula:

%FAW infestation = (Number of FAW infested plants)/(Total number of plants observed) × 100

Leaf damage was scored by visual observation using the scoring scale of 0–9 reported by Davis and Williams [13] (Table 2). Leaf damage scores of individual plants in quadrants of each surveyed farm were averaged to determine the leaf damage ratings of a district.

### 2.3. Assessment of Natural Enemies of FAW

Surveys of natural enemies were conducted in Ethiopia from March to October in 2017, and in Kenya and Tanzania from July 2017 to November 2017. In Ethiopia and Kenya, surveys of FAW natural enemies were also conducted from June to August 2018. A total of 101 farms in Ethiopia, 21 farms in Kenya and 13 in Tanzania were sampled. Location details such as latitude, altitude and longitude were taken using GPS. In each surveyed farm, three quadrants measuring 3 m × 3 m were randomly selected. The number of egg masses and larvae were counted on the damaged maize plants. The egg masses were placed in plastic cups with about 5 g of natural diet (fresh maize leaf). Upon hatching, the larvae were placed in rectangular plastic cages (4 cm height × 15 cm width × 21 cm length), covered on top with a fine screen to prevent the escape of parasitoids. The larvae were fed with around 60 g of maize shoot, replaced every 48 h, until pupation.The eggs and larvae were kept in the laboratory at room temperature of 24–26 °C, 50–70% RH and a photoperiod of 12:12 (L:D) hour until parasitoids emerged. The parasitoids that emerged from the eggs or larvae were recorded every 24 h until pupation. No dissections of dead eggs or larvae were made to examine for dead parasitoids [13,15]. Parasitoids were preserved in 70% ethanol and sent for identification to the Natural History Museum, UK. We did not find any occurrences of multiple parasitism in this study. Percent parasitism was calculated according to Pair et al. [16].

% Parasitism = (Number of parasitoids)/(Number of larvae collected) × 100

### 2.4. Data Analysis

Percent fall armyworm-infested fields, percent fall armyworm infestation, leaf damage score (scale 1–9) and percent parasitism of natural enemies were summarised and descriptive statistics (means and percentages) were calculated. All statistical analysis was done using MINITAB 16 statistical software.

## 3. Results

### 3.1. Distribution and Damage by FAW

FAW is widely distributed across maize-growing districts of Ethiopia, Kenya and Tanzania (Table 3 and Table 4) and was present in most fields surveyed. In 2017, the percentage of infested fields ranged from 33% to 100% in Ethiopia, 93% to 100% in Tanzania and was 100% for the farms observed in Kenya (Table 3). In Ethiopia, the highest infestation was found in Shebe Senbo (62.3%), whilst the lowest infestation was recorded in Bahir Dar (5.7%). In Kenya, relatively high percentages of FAW infestation were observed, ranging from 77% in Mt Elgon to 100% in Webuye East and Tongaren in 2017. In Tanzania, Morogoro and Kilombero showed 72.7% and 95.7% infestation, respectively. Leaf damage score ranged from 1.8 to 7 in Ethiopia, 3.2 to 5.3 in Kenya and 3.7 to 5.2 in Tanzania in 2017 (Table 3).

In 2018, the percentage of FAW-infested fields ranged from 80% to 100% in Ethiopia and Kenya. In Ethiopia, the highest percentage infestation was recorded in Metehara (49.3%), while the lowest was in Lock Abaya (4.9%). In Kenya, the highest percentage infestation was recorded in Masheni (96.2%), while the lowest was in Kepkelion (50.4%). In Ethiopia, maize leaf damage score ranged from 1.9 to 5.4, whereas in Kenya, it ranged from 5.0 to 6.8 (Table 4).

### 3.2. Recruitment of Local Parasitoids by FAW

**Figure 2:**
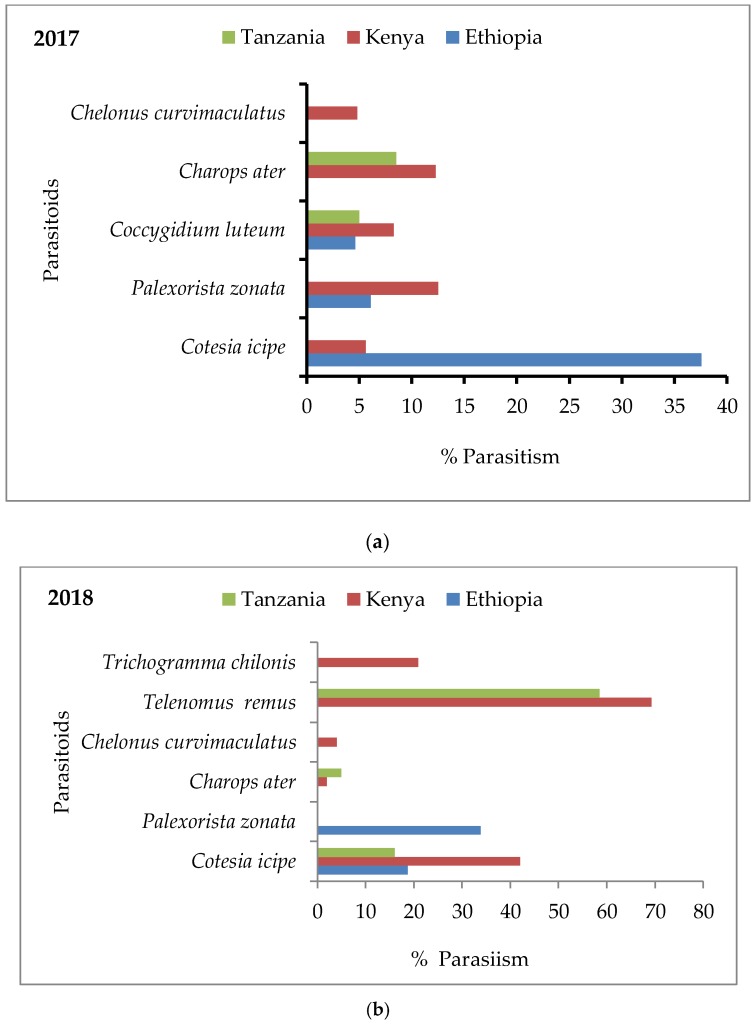
Percent occurrence of FAW parasitoids collected from Ethiopia, Kenya and Tanzania in (**a**) 2017 and (**b**) 2018.

In 2017, in Ethiopia, Kenya and Tanzania, five different species of parasitoids belonging to Hymenoptera and Diptera were recovered from FAW eggs and larvae (Figure 2). In Ethiopia, *Cotesia icipe* Fernández-Triana & Fiaboe (Hymenoptera: Braconidae) was the main larval parasitoid, with 37.6% parasitism, followed by *Palexorista zonata* (Curran) (Diptera: Tachinidae) and *Coccygidium luteum* (Brullé) (Hymenoptera: Braconidae), with 6.1% and 4.6% parasitism, respectively. In Kenya, one species of egg-larval parasitoid and four species of larval parasitoids were recorded. *Palexorista zonata* was the dominant parasitoid, with 12.5% parasitism, followed by *Charops ater* Szépligeti (Hymenoptera: Ichneumonidae) and *C. luteum*, with 12.3% and 8.3% parasitism, respectively. In addition, *Chelonus curvimaculatus* Cameron (Hymenoptera: Braconidae) was the only egg-larval parasitoid recorded in Kenya and had low levels of parasitism (4.8%). In Tanzania, only two species of larval parasitoids were recovered, namely *C. ater* and *C. luteum*, causing 8.5% and 5% parasitism, respectively.

In 2018, in Ethiopia, Kenya and Tanzania, six different species of parasitoids were found to be associated with eggs and larvae of FAW (Figure 2). In Ethiopia, two species of larval parasitoids were recovered, namely *P. zonata* and *C. icipe*, which accounted for 33.9% and 18.7% of parasitism, respectively. In Kenya, *Telenomus remus* (Hymenoptera: Scelionidae) was the dominant egg parasitoid and accounted for 69.3% of egg parasitism, followed by *Trichogramma chilonis*, which accounted for 20.9% of egg parasitism, whereas *Chelonus curvimaculatus* caused low levels of egg parasitism (4%). *Cotesia icipe* was the main larval parasitoid, which caused 42% parasitism, whilst *C. ater* caused low levels of parasitism (2%). In Tanzania, *Telenomus remus* was the dominant egg parasitoid, causing 58.5% parasitism. *Cotesia icipe* caused 16% parasitism, whereas *C. ater* caused low levels of parasitism (5%) (Figure 2).

## 4. Discussion

Fall armyworm has rapidly spread throughout most of the maize-producing areas of Ethiopia, Kenya and Tanzania, as suggested by the moderate to high levels of infestation of FAW in almost all maize fields surveyed. Following the first report of FAW in West Africa in January 2016, the pest was soon found in East Africa in 2017 [2,3]. Its modality of introduction, along with its ecological adaptation and spread across Africa, is still speculative. However, wind-assisted flight, hidden infestations in trade commodities and human-assisted transport have been implicated as likely mechanisms for facilitating the rapid spread of the pest [3]. Percent FAW infestation varied considerably among the farms we surveyed in the three countries, with mean percent FAW infestation ranging from 5.3% to 100%. Relatively high percentages of FAW infestation (>73%) were recorded in Kenya and Tanzania. In addition, most of the farms surveyed had low to moderate leaf damage scores. In Ethiopia, high mean percent FAW infestation was recorded in Semen Bench (78.7%), Shebe Senbo (62.2%) and Adama (60.2%) in 2017. High mean percent FAW mortality (49.25%) was recorded in Dedo in 2018. Although larvae feed on both vegetative and reproductive maize [2,10], feeding on leaf tissue only may not cause yield loss as the plant can tolerate such damage. Subsequent yield loss also depends on the growth stage of maize and level of infestation [2].

In this study, a total of five species of parasitoids were recorded from FAW eggs and larvae collected in Ethiopia, Kenya and Tanzania. Results of this study are consistent with recent findings of Sisay et al. [17], who reported that *C. icipe* was the dominant larval parasitoid, with parasitism ranging from 33.8% to 45.3% in Ethiopia. However, in the present study, percent of parasitism by *C. icipe* was lower in Ethiopia, but higher in Kenya as compared to the previous year’s results reported by Sisay et al. [17]. In Kenya, the Tachinid fly, *P. zonata*, was the primary parasitoid, with 12.5% parasitism. *Charops ater* and *C. luteum* were the most common parasitoids in Kenya and Tanzania, with parasitism ranging from 6% to 12% and 4% to 8.3%, respectively [17]. Furthermore, two additional egg parasitoids, namely *Telenomus remus* and *Trichogramma chilonis*, were recovered in 2018, with high levels of egg parasitism found in *Telenomus remus*. Those parasitoids found in the present study have been also recovered from FAW eggs and larvae in East and West Africa [18]. Recently, Kenis et al. [19] reported the presence of *T. remus* in different countries in Africa. Furthermore, some of the parasitoids recovered in this study have been reported as parasitising other insect species; for example, *C. icipe*, which is a new species from eastern Africa, was reared in Kenya as a solitary parasitoid from *Spodoptera littoralis* (Boisduval, 1833) and *S. exigua* (Hübner, 1808) [20]; further, *Charops ater* was reported to parasitise African bollworm and other species in Kenya [21]. In addition, *P. zonata* was recorded from the African armyworm, *Spodoptera exempta*, by Rose et al. [22] indicating the recruitment of native parasitoids to FAW in eastern Africa. Variations in parasitoid species composition and the level of parasitism may be due to differences in geographical locations, agronomic practices and crop type and stage [16,23]. In North and South America, which are native regions of FAW, various species of natural enemies attacking FAW have been documented. For example, Molina-Ochoa et al. [13] reported a total of 150 species of parasitoids of FAW. This may indicate that while emphasis should be given to local surveys of native natural enemies, introduction of effective natural enemies from the Americas through classical biocontrol can be an option when there is a gap [5,20].

FAW is the most difficult pest to control due to its multiple generations, ability to migrate and ability to feed on a wide range of host plants. To tackle the menace of the fall armyworm pest to avoid economic adversity for smallholder farmers in Africa, rapid and coordinated action, enormous awareness creation, technological innovation and national, regional and international collaborations are required. Biological control can play a significant role in an integrated management of FAW to provide sustainable solutions to effectively tackle the adverse effects of this pest. The current study, therefore, contributes to the management of the FAW in identifying effective natural enemies that could be used in the integrated management of FAW. The new associations of various species of natural enemies with FAW in Africa across countries and season in the current study shows the paramount importance of designing biological controls of FAW both through the conservation of native natural enemies and augmentative release. The present massive application and indiscriminate use of pesticides in Africa against FAW might have negative impact on the natural enemies; hence, it is vital to protect natural enemies from adverse effects of pesticide and design IPM strategies for FAW management in the region.

## 5. Conclusions

In conclusion, the present study confirms the rapid and substantial expansion of the FAW range in eastern Africa. Native species of parasitoids recovered from eggs and larvae of FAW are crucial for implementing biological control programmes of this pest, which are essential components in developing integrated pest management approaches for FAW in eastern Africa.

## Figures and Tables

**Table 1:** Survey districts in Ethiopia, Kenya and Tanzania.

Country	District	GPS Record
Ethiopia	Shebe Senbo	7.464N, 36.4219E
	Dedo	7.613533333N, 36.83481667E
	Seka Chekorsa	7.56465N, 36.64643333E
	Debub Bench	6.925316667N, 35.50806667E–6.926083333N, 35.51278333E
	Semen Bench	7.673166667N, 35.7101E
	Lock Abaya	6.6757N, 38.26178333E
	Hawassa	7.000421667N, 38.38775E–7.019233333N, 38.37613333E
	Bahir-Dar	11.6815N, 37.4575E–11.69873333N, 37.48608333E
	Jabitenan	10.56158333N, 37.1779E–10.69175N, 37.17113333E
	Bure	10.70236667N, 37.10995E–10.70781667N, 37.11526667E
	Adama	8.414033333N, 39.32258333E–8.420366667N, 39.32061667E
	Metehara	8.637966667N, 39.41063333E–8.8016N, 39.89383333E
Kenya	Webuye East	0.5877N, 34.75556667E
	Tongaren	0.84182N, 35.00448333E
	Mt. Elgon	0.50456N, 34.4345E
	Kabuchai	0.36023N, 34.37336667E
	Kipkelion East	−0.206816667N, 35.56015E
	Gilgil	−0.521383333N, 36.09068333E
Tanzania	Kilombero	−7.42583N, 36.98886667E– −7.557N, 37.01718333E
	Morogoro	−6.2149N, 37.57918333E– −6.39344N, 37.5582E

**Table 2:** Visual rating scales for leaf damage assessment [14].

Scale	Description
0	No visible leaf damage
1	Only pinhole damage on leaves
2	Pinhole and shot hole damage to leaf
3	Small elongated lesions (5–10 mm) on 1–3 leaves
4	Midsized lesions (10–30 mm) on 4–7 leaves
5	Large elongated lesions (>30 mm) or small portions eaten on 3–5 leaves
6	Elongated lesions (>30 mm) and large portions eaten on 3–5 leaves
7	Elongated lesions (>30 cm) and 50% of leaf eaten
8	Elongated lesions (30 cm) and large portions eaten on 70% of leaves
9	Most leaves with long lesions and complete defoliation observed

**Table 3:** Mean percent of fields infested by fall armyworm (FAW) and level of infestation in different survey districts of Ethiopia, Kenya and Tanzania in 2017.

Country	District	% Infested Fields	% FAW Infestation	Leaf Damage Ratings (Scale 1–9)	Number of Fields
Ethiopia	Dedo	81	33.8 ± 5.8	3.5 ± 0.524	27
	Seka Chekorsa	69	15.8 ± 5.66	2.1 ± 0.515	16
	Shebe Senbo	100	62.3 ± 6.88	5.7 ± 0.518	13
	Debub bench	33	44.4 ± 23.4	4.3 ± 2.33	3
	Semen bench	100	78.7± 0.0	7.0 ± 0	1
	Lock Abaya	100	30.0 ± 11.9	4.0 ± 1.15	3
	Hawassa	100	43.7 ± 10.7	4.8 ± 0.701	8
	Bahir Dar	100	5.3 ±1.09	1.8 ± 0.200	6
	Jabitenan	100	13.9 ± 6.49	2.5 ± 0.50	4
	Bure	100	6.7 ± 0.98	2.0 ± 0.00	2
	Adama	100	60.2 ± 7.03	5.0 ± 0.408	4
Kenya	Webuye East	100	100.0 ± 9.51	3.5 ± 0.130	20
	Tongaren	100	100.0 ± 0.0	3.2 ± 0.519	4
	Kabuchai	100	91.0 ± 1.83	3.9 ±0.246	16
	Mt Elgon	100	77.0 ± 3.04	3.8 ±0.300	9
	Kipkelion East	100	86.0 ± 2.09	4.6 ±0.399	8
	Gilgil	100	91.0 ± 3.66	5.3 ± 0.894	3
Tanzania	Morogoro	93	72.7 ± 4.71	3.7 ± 0.384	10
	Kilombero	100	95.7± 95.1	5.2 ± 0.648	8

**Table 4:** Mean percent of fields infested by FAW and level of infestation in different survey districts of Ethiopia and Kenya in 2018.

Country	District	% Infested Fields	% FAW Infestation	Leaf Damage Ratings (Scale 1–9)	Number of Fields
Ethiopia	Dedo	86.7	28.6 ± 6.55	3.7 ± 0.25	12
	SekaChekorsa	93.3	20.2 ± 6.95	3.5 ± 0.50	15
	ShebeSenbo	80.0	9.9 ± 1.64	2.5 ± 0.56	15
	Lock Abaya	90.0	4.9 ±1.32	1.9 ± 0.15	10
	Hawassa	92.0	26.5 ± 5.98	4.1 ± 0.70	26
	Adama	100.0	11.0 ± 2.17	2.7 ± 0.56	7
	Metehara	100.0	49.3 ± 10.96	5.4 ± 0.40	16
Kenya	Tongaren	100.0	74.0 ± 2.67	6.4 ± 0.125	3
	Kabuchai	100.0	69.3 ± 3.25	5.2 ± 0.15	2
	Webuye	100.0	71.0 ± 2.5	6.5 ± 0.25	4
	Elgon	100.0	66.2 ± 2.57	5.0 ± 0.3	3
	Kepkelion Masheni	94.0 82.2	50.4 ± 1.9 96.2 ± 3.78	4.8 ± 0.05 6.8 ± 0.17	3 6
